# Impact of oral health on frailty syndrome in frail older adults

**DOI:** 10.31744/einstein_journal/2023AO0103

**Published:** 2023-07-31

**Authors:** Maria Cecilia Ciaccio Vendola, Wilson Jacob-Filho

**Affiliations:** 1 Hospital das Clínicas Faculdade de Medicina Universidade de São Paulo São Paulo SP Brazil Hospital das Clínicas, Faculdade de Medicina, Universidade de São Paulo, São Paulo, SP, Brazil.

**Keywords:** Frail elderly, Frailty, Oral health, Health status, Aging, Self report, Surveys and questionnaires

## Abstract

**Objective:**

This study aimed to correlate oral and general health in frail and non-frail older adults.

**Methods:**

This observational study included 52 older adults, of whom 35 were frail (Frail Group), and 17 were non-frail (Non-Frail Group), according to Fried’s self-reported test addressing oral health variables, number of systemic diseases, and medications in use. The geriatric oral health assessment index was used to assess the oral hygiene of the groups.

**Results:**

The number of preserved teeth in dentulous older adults was significantly higher in the Non-Frail Group (p=0.048). No significant differences were observed between the two groups in the use of dental prostheses or in the detection of soft tissue lesions. Overall, 74.3% of the Frail Group had a “bad” geriatric oral health index score, which significantly differed from that of the Non-Frail Group (p=0.045). The numbers of systemic diseases and medicines used were higher in the Frail Group than in the Non-Frail Group (p<0.001), demonstrating the pathophysiological characteristics of multimorbidity and polypharmacy in frailty syndrome.

**Conclusion:**

The results showed a clear correlation between oral and general health conditions and frailty syndrome.

**Figure:**
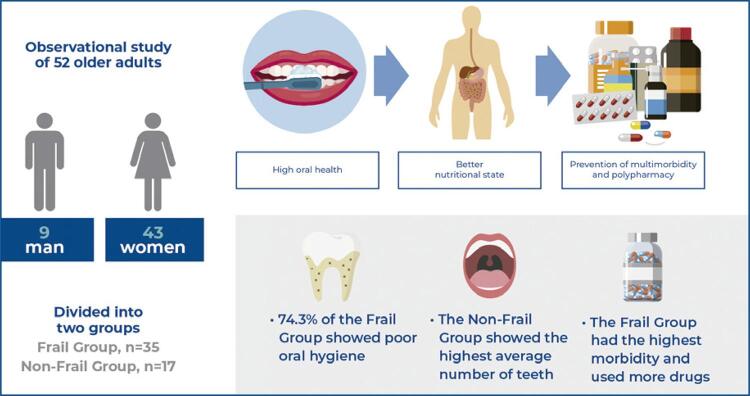

## INTRODUCTION

### Aging population

In the global context, the aging population increased slowly in the first half of the 20^th^ century, increased more rapidly in the second half, and has been greatly accelerating in the early 21^st^ century. In 2019, the United Nations epidemiology division published a new global population projection. The number of Brazilian adults aged 60 years or older has risen to 29.9 million in 2020, whereas the number of adults aged 65 years or older worldwide has risen to 422 million in 2020 (6.5%). The worldwide population is expected to reach 2.5 billion by 2100, projected to be 80 years old, and 72 million more by 2020 and to reach 881.^(1)^

Older adults will be influencing guidelines regarding their health and the economic sustainability of this phenomenon. The impact of multimorbidity characterizing senility and the consequent use of healthcare services and their associated costs are often significant factors affecting the availability of necessary resources and the potential effects of these interventions.^(2)^

### Health in aging

Population studies associating the aging process with genetic factors have shown at least two sociodemographic factors that influence longevity: fertility in men, who do not undergo the physiological changes that women do (climacteric), and selection of alleles that extend life expectancy in both sexes.^(3)^

The development of care involves a positive genetic correlation between longevity and the attitudes of relatives who provide care, facilitating greater longevity.^(4)^

Aging science is improving and promoting changes in human habits and behaviors in favor of healthy aging, but in parallel with an intense trend toward urbanization, triggering stressors that compromise health at all ages and increase the incidence and aggressiveness of illnesses. Owing to these factors, studies using demographic and ecological models to predict the impact of the aging population are necessary in this decade, as older adults (80 years or older) are increasing in number exceedingly.^(5)^

Several studies have correlated environmental and lifestyle changes with the quality of aging; however, these studies also need to extend to determinants of oral health, including hygiene care, teeth condition, preservation of masticatory mechanics, esthetics, and structure. Aging leads to a greater vulnerability to internal (intrinsic capacity) and external (extrinsic capacity) factors that predispose individuals to increased morbidity and mortality. In this context, the frailty syndrome can develop, which is the central theme of this study.

### Frailty syndrome

Older adults experience signs and symptoms that are predictors of several future health complications, making it an important public health problem. The term “frail elderly” was introduced by Monsignor Charles F. Fahey and members of the Federal Council on Aging of the USA in the 1970s, when an individual who lived in unfavorable socioeconomic conditions and presented physical and cognitive weaknesses was considered frail.^(6)^

At the end of the 1980s, other studies were conducted in which “frail elderly” people were described as individuals aged 65 years or over, with dependency for activities of daily living, and affected by comorbidities. In 1990, the first reference to “frail elderly” people appeared in the index of the Journal of the American Geriatrics Society. During the 1990s, the term frailty was increasingly studied, with the question of whether frailty is synonymous with incapacity.^(7)^

Several studies have stated that frailty is directly related to the presence of some diseases, such as stroke and chronic non-communicable diseases, leading to numerous functional disabilities, including mental confusion, immobility, addiction, depression, falls, incontinence, malnutrition, polypharmacy, pressure injuries, and sensory problems, with clear socioeconomic sequalae.^(8,9)^

Various methods can be used to track and identify different degrees of frailty. In consensus, frailty in older adults is characterized by symptoms such as unintentional weight loss (5kg in the last 5 years), self-reported fatigue, reduced physical capacity, slowing of movements, and reduced social activity.^(7)^ Frailty encompasses physical, psychological, and social multidimensional domains, as stated by Gobbens et al.^(10)^ and is currently considered a syndrome.

### Oral health

The propaedeutic peculiarities inherent in the oral health of older adults result from knowledge about the human aging process and its nuances from an interdisciplinary perspective; thus, dentists must be knowledgeable about more than techniques and materials.^(11)^

The oral health condition is evaluated using clinical criteria, the individual’s self-perception, and its impact on quality of life.^(12)^ Therefore, frailty in older adults, which shows a greater burden of systemic diseases and use of medications, should be regarded through the prospective coexistence with poorer oral health, leading to a lower quality of life.^(13)^

Owing to the aging population and increase in the number of people using different medications, dentists may encounter xerostomia more frequently. This symptom may be associated with difficulties in chewing, swallowing, tasting (dysgeusia), alteration of smell (halitosis), and phonation. It can result in malnutrition and decreased social interactions and discomfort in the mouth, especially in individuals wearing dentures, particularly full dentures. Patients with xerostomia also have an increased risk of developing tooth decay because decreased saliva facilitates biofilm formation and more active invasive bacterial action.^(14,15)^

The growing space that geriatric dentistry occupies in the context of hospital dentistry comes from the progressive needs of older adult patients and is based on the degree of fragility and the individual evolution of the systemic impairments. Dental care is highly complex in this scenario. In these cases, the dental surgeon can use the hospital structure for more invasive dental procedures and ensure adequate patient safety. The classification of the degree of systemic complexity in older patients can be based on the system of the American Society of Anesthesiologists (ASA). Among older patients who are ASA III (severe systemic disease, in which patients are limited but not incapacitated), using the hospital structure for higher-risk dental interventions is safe.^(16)^

The World Health Organization (WHO)^(2)^ states that oral diseases can affect the performance of an individual, either at school or at work; that they can cause social and personal problems, whose psychosocial impact substantially reduces the quality of life; and that oral alterations tend to disproportionately affect the poorest and most socially disadvantaged population in their aging process. According to the WHO,^(2)^ oral health is defined as the absence of pain, either in the mouth or face, cancer of the mouth and/or throat, oral infections and lesions, and gum and dental diseases. Limiting factors include psychosocial behavior, well-being, and the ability to bite, chew, smile, and speak.

Oral health professionals for older adult patients should surpass the principles of rehabilitation (fundamental to the beginning of the digestive process) and contemplate the issues of bacterial plaque, oral hygiene, and risks of periodontal disease. The anaerobic bacteria, especially *Porphyromonas gingivalis*, has recently been studied as a factor involved in the gradual formation of amyloid proteins in brain regions, contributing to the development of Alzheimer’s disease.^(17)^

Chronic obstructive respiratory disease is also associated with a lack of oral hygiene and is a risk factor among older adults.^(18,19)^

During the coronavirus disease 2019 (COVID-19) pandemic, oral antimicrobial mouthwashes, such as chlorhexidine, were ineffective against the new coronavirus. However, in more severe viral respiratory diseases, such as acute respiratory syndrome and middle east respiratory syndrome, which also cause many hospitalizations, oral healthcare is considered essential.^(20)^

In Brazil, edentulism represents the legacy of a dental model centered on non-conservative curative procedures, which has resulted in many extractions and a high demand for prosthetic services.^(21)^

## OBJECTIVE

To study the related oral and general health conditions of frail and non-frail older adults and their impact on their life.

## METHODS

This observational cross-sectional study was approved by the Research Ethics Committee of *Hospital das Clinicas* of the *Faculdade de Medicia* of the *Universidade de São Paulo* (CAAE: 82201518.6.0000.0068; #2.694.474). This study was conducted with a sample of 52 participants from the outpatient clinics of the Geriatrics Service of the *Hospital das Clinicas, Universidade de São Paulo* (Fragility Ambulatory and Ambulatory of the Multidisciplinary Care Group for the Older Adults Outpatient - GAMIA) (Table 1). Inclusion in the fragile group (FG) was based on a positive answer to three or more questions of the self-reported Fried test, and inclusion in the non-FG (NFG) was based on no positive answers to the same instrument. All participants signed the informed consent form to participate in the research.

**Table 1:** Sex and age of the participants

	Total n (%)	Frail Group n (%)	Non-Frail Group n (%)	p value
Older adults	52	35 (67.3)	17 (32.6)	
Male sex	9 (17.3)	7 (20)	2 (11.7)	
Female sex	43 (82.7)	28 (80)	15 (88.3)	
Female/male		4	7.5	
Average age (years)		84.80	81.20	0.085^†^

^†^ Fisher’s exact test.

This study was conducted at the Frail Outpatient Clinic of the Geriatrics Service of the *Hospital das Clínicas da Faculdade de Medicina* of the *Universidade de São Paulo* using the self-reported Fried test,^(22,23)^ which comprises four questions related to events occurring in the past year (weight loss, reduction in strength, reduction in walking speed, and reduction in motor activities) and a question about fatigue related to the past week. To better assess the relationship between oral health conditions and their impact on quality of life in this study, the Geriatric Oral Health Assessment Index (GOHAI), an instrument proposed by Atchison et al,^(24)^ was administered, as the original version has been shown to have high internal consistency and reliability. It comprises 12 questions related to the past 3 months, covering physical, psychosocial, and pain dimensions.

In the study, interviews with each participant were conducted by a single researcher in a single session from both outpatient clinics. The order of collection of data during the interview was as follows. Self-reported questions^(22)^ assessed the degree of frailty in the older patients, with 1 point being allocated for affirmative answers. Those who scored 0 points after answering the five questions were classified into the NFG and those who scored 3 or more points were classified into the FG. Oral health data collection was performed through specific and standardized dental clinical examinations in both groups,^(24)^with three possible answers (always, sometimes and never) that were scored 1, 2 and 3, respectively. The scores for questions 3, 5, and 7 were reversed (3, 2, and 1, respectively) for easier comprehensibility. The sum of the points was classified as great (34–36), regular (30–33), or bad (29 or less). The electronic medical records were accessed for data collection concerning disease history and medication use in the consultation prior to the evaluation for guidance.

The data were tabulated in an Excel spreadsheet, and the clinical examination of the oral cavity was standardized and divided into the following variables: number of teeth (NT), use of maxillary total denture, use of total mandibular denture, use of removable upper partial prosthesis (URPP), use of removable lower partial prosthesis (LRPD), use of conventional fixed prosthesis (CFP), use of implant prosthesis (PI), and presence of soft tissue injuries (STIs).

From the electronic medical records, updated information was collected from the most recent consultation for each patient for the following variables: numbers of systemic diseases and medications used.

## RESULTS

After 88 patients were screened and evaluated according to the degree of frailty, the study sample included 52 adults (FG: 35 and NFG: 17) of both sexes aged 60 years or older. Although the percentage of edentulism was similar in both groups (FG: 57.2% and NFG: 58.8%), the mean NT was significantly higher in the NFG than in the FG (Table 2).

**Table 2:** Number of teeth and edentulism

	Frail Group n (%)	Non-Frail Group n (%)	Total n (%)	p value
Average number of teeth	10.86	20.14		0.048^#^
Edentulism	20 (57.2)	10 (58.8)	30 (57.7)	
Teeth	15 (42.8)	7 (41.2)	22 (42.3)	

^#^ Mann-Witman test.

**Figure 1:**
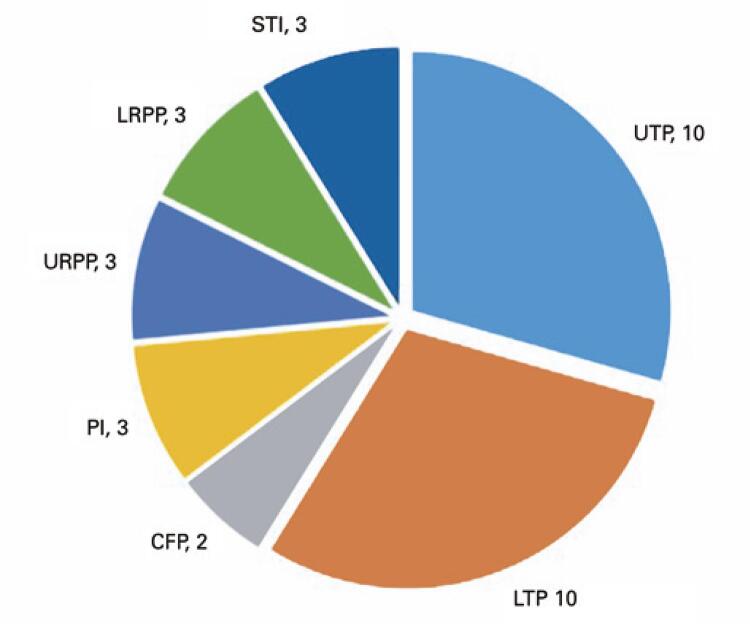
Non-Frail Group - prostheses distribution and soft tissue injuries

UTP: upper total prosthesis; LTP: lower total prosthesis; URPP: upper removable partial prosthesis; LRPP: lower removable partial prosthesis; CFP: conventional fixed prosthesis; PI: implant prosthesis; STI: soft tissue injury.

**Figure 2:**
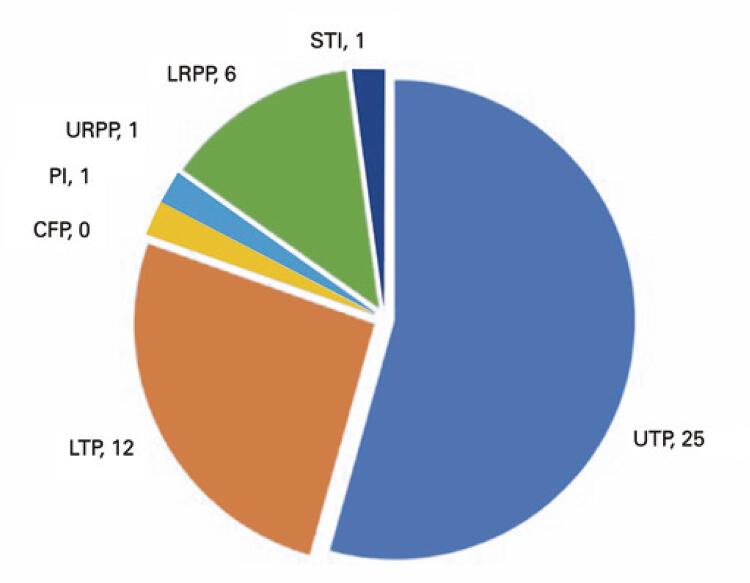
Frail Group - prostheses distribution and soft tissue injuries

UTP: upper total prosthesis; LTP: lower total prosthesis; URPP: upper removable partial prosthesis; LRPP: lower removable partial prosthesis; CFP: conventional fixed prosthesis; PI: implant prosthesis; STI: soft tissue injury.

With regard to the use of CFPs or PIs, URPP or LRPD, and STIs, the statistical analysis showed no significant differences between the groups (Table 3), although there seemed to be an interesting diversity in the distribution of teeth in the upper and lower arches, as observed by the total and removable prostheses (Figures 1 and 2).

**Table 3:** Presence of prostheses and soft tissue lesions

Variable	Frail Group n=35	Non-Frail Group n=17	Total n=52	p value
UTP	25 (71.4)	10 (58.8)	35 (67.5)	0.529^†^
LTP	12 (34.3)	10 (58.8)	22 (42.3)	0.136^†^
URPP	1 (2.9)	3 (17.6)	4 (7.7)	0.097^†^
LRPP	6 (17.1)	3 (17.6)	4 (7.7)	1.0^†^
CFP	0	2 (11.8)	2 (3.8)	0.136^†^
PI	1 (2.9)	3 (17.6)	4 (7.7)	0.097^†^
STI	1 (2.9)	3 (17.6)	4 (7.7)	0.097^‡^

† χ^2^ test; ^‡^ Student’s *t* test.

UTP: upper total prosthesis; LTP: lower total prosthesis; URPP: upper removable partial prosthesis; LRPP: lower removable

partial prosthesis; CFP: conventional fixed prosthesis; PI: implant prosthesis; STI: soft tissue injury.

In the GOHAI results, three-fourth of the FG presented with a “bad” score (poor hygiene, fewer teeth, and negative self-image), whereas more than two-third of the NFG present with a “regular” or “great” score. The results were considered statistically significant at p=0.045. Considering the three dimensions (physical, psychic, and pain), we observed that the differences were mainly in the first two, with a significantly better evaluation in the FNG regarding their perceptions related to their esthetics and social interactions (Table 4).

**Table 4:** Geriatric oral health assessment index and dimensions

Score	Frail Group n=35	Non-Frail Group n=17	Total n=52	p value
Great	2 (5.7)	5 (29)	7 (134)	
Regular	7 (20)	6 (35.5)	13 (25)	
Bad	26 (74.3)	6 (355)	32 (61.5)	
				0.045^†^
Dimensions - average				
Physical	8.5	10.6	9.2	0.002^‡^
*Psychic	8.9	10.2	9.3	0.045^‡^
Pain	8.6	8.8	8.7	0.82^‡^

^†^ χ^2^ test; ^‡^ Student *t* test.

* Psychosocial can also be used. Great from 34 to 36, regular from 30 to 33, bad from 29 or less.

The results of the assessment of general health status based on the number of systemic diseases and medication use were favorable for the NFG, with approximately 40% of the number of diagnoses and approximately 50% of medication use in relation to those of the FG (Table 5).

**Table 5:** Systemic diseases and medications used

	Frail Group	Non-Frail Group	Total	p value
Systemic diseases number	7.9	3.1	6.3	<0.001^#^
Medications number	6.4	3.2	5.3	<0.001^#^

^#^ Mann Witman test.

## DISCUSSION

Given the global reality of population aging, the greatest challenge for health systems in the 21^st^ century is to prevent the occurrence of early fragility syndrome resulting from a poorly structured aging process. Therefore, there is a constant demand for prevention and early detection of this evolution to avoid disastrous evolution as much as possible. In this search, oral health was rarely included in the global assessment of older adults and was even less identified as a determinant factor of the global health of the older population. However, the literature offers important information on the enormous influence of the conditions of the oral cavity and its attached structures, which are directly or indirectly related to systemic health and diseases that affect individuals during the course of life.^(25)^

Edman et al.^(26)^ evaluated 273 older people twice and 10 years apart, noting that risk factors such as poor dental hygiene, with only one brushing daily and little use of dental floss, increased the incidence of carious lesions in one-fourth of this population, in addition to tooth loss of two teeth on average per individual during this period, thus reinforcing the importance of correct daily hygiene measures for “frail elderly” people who need caregivers to collaborate with their oral hygiene.

Singhrao et al.^(27)^ stated that chronic periodontitis can become a risk factor for the incidence of Alzheimer’s Disease, demonstrating the importance of oral health for the systemic health of older adults.

Dantas et al.^(12)^demonstrated that the lack of teeth has a significant negative impact on the quality of life of edentulous older adults. In our study, the frequency of edentulism was very high in both groups, which can be explained by the precocity with which total tooth extraction was performed in the past, especially among those with lower purchasing power.^(26)^The determinant factors for this mutilation were not the impairment of health but the precariousness of dental care. The NT in the other groups, however, showed a clear difference in favor of NFG, revealing that healthier teeth were more likely to preserve their natural dentition (Table 2).

Similarly, we did not observe significant differences in the use of dentures, considering that edentulous patients only had the option of complete dentures or dentures over implants (Table 3; Figures 1 and 2).

Strauss et al., carried out a study with older adults to determine how teeth affect the perception of health, and showed that poor dental condition can cause harm to the physical condition and quality of life due to the loss of chewing capacity, comfort in daily life, and low self-esteem.^(8)^ This becomes even more relevant throughout the aging process, where the risks of frailty can be cumulative as a result of the quality of life and satisfaction with it.^(23)^ In our study, the evaluation of the GOHAI showed interesting results that were in agreement with the characteristics of each group. The score was predominantly in the great and regular groups in the NFG, while it was prevailing in the bad group in the FG, mainly due to the differences in scores in the physical and psychiatric dimensions, in our view, the most affected by poor oral health and Frailty Syndrome.

Saliva has been the subject of many studies because it contains large amounts of biochemical elements that are important in the digestive process and in the feeling of well-being through breathing and phonation. Its reduction, either due to the use of a greater amount of medication or due to the greater number of systemic diseases that characterize Frailty Syndrome, was associated with a poor oral cavity condition.^(28)^ Physical exercise is also associated with the maintenance of good salivary levels^(4,9)^ in addition to preventing the harmful consequences of pathological aging, protecting the cardiovascular system, and contributing to a better quality of life. Peng et al.^(29)^ revealed that the ACE2 receptor is found in the epithelial cells of the salivary gland ducts, the gateway to COVID-19, attributing the power to oral secretions to spread it quickly throughout the body and environment, confirming the association of poor oral hygiene, more disease diagnoses, and the use of a greater number of oral medications in the FG. With the continuous daily use of these two medications, older adults are already subject to a decrease in saliva in the oral cavity.^(15)^

According to Niesten et al.^(30)^ frail older adults show significant improvement in self-esteem when they manage to maintain or restore their teeth in a good condition, as tooth removal becomes an aggravating factor for depressive symptoms related to negative self-image. In our opinion, these data point to a strong relationship between oral and general health in terms of its multiple physical, psychological, and social components. They also show that the correction of a health deviation will have an enormous benefit that will not be restricted to the treated segment but will affect the entire systemic universe of those who get older.

## CONCLUSION

This study demonstrates that good oral health condition leads to a reduced risk for developing diseases in aging and collaborate with the preservation of self-image and social interaction for a better quality of life.
